# Tetracycline Inducible Gene Manipulation in Serotonergic Neurons

**DOI:** 10.1371/journal.pone.0038193

**Published:** 2012-05-31

**Authors:** Tillmann Weber, Insa Renzland, Max Baur, Simon Mönks, Elke Herrmann, Verena Huppert, Frank Nürnberg, Kai Schönig, Dusan Bartsch

**Affiliations:** 1 Department of Molecular Biology, Central Institute of Mental Health, Medical Faculty Mannheim/Heidelberg University, Mannheim, Germany; 2 Department of Addictive Behavior and Addiction Medicine, Central Institute of Mental Health, Medical Faculty Mannheim/Heidelberg University, Mannheim, Germany; 3 Department of Psychiatry and Psychotherapy, Central Institute of Mental Health, Medical Faculty Mannheim/Heidelberg University, Mannheim, Germany; 4 Institute for Applied Mathematics, Faculty for Informatics, Mannheim University of Applied Sciences, Mannheim, Germany; Max-Delbrück Center for Molecular Medicine (MDC), Germany

## Abstract

The serotonergic (5-HT) neuronal system has important and diverse physiological functions throughout development and adulthood. Its dysregulation during development or later in adulthood has been implicated in many neuropsychiatric disorders. Transgenic animal models designed to study the contribution of serotonergic susceptibility genes to a pathological phenotype should ideally allow to study candidate gene overexpression or gene knockout selectively in serotonergic neurons at any desired time during life. For this purpose, conditional expression systems such as the tet-system are preferable. Here, we generated a transactivator (tTA) mouse line (TPH2-tTA) that allows temporal and spatial control of tetracycline (Ptet) controlled transgene expression as well as gene deletion in 5-HT neurons. The tTA cDNA was inserted into a 196 kb PAC containing a genomic mouse *Tph2* fragment (177 kb) by homologous recombination in *E. coli*. For functional analysis of Ptet-controlled transgene expression, TPH2-tTA mice were crossed to a Ptet-regulated lacZ reporter line (Ptet-nLacZ). In adult double-transgenic TPH2-tTA/Ptet-nLacZ mice, TPH2-tTA founder line L62-20 showed strong serotonergic β-galactosidase expression which could be completely suppressed with doxycycline (Dox). Furthermore, Ptet-regulated gene expression could be reversibly activated or inactivated when Dox was either withdrawn or added to the system. For functional analysis of Ptet-controlled, Cre-mediated gene deletion, TPH2-tTA mice (L62-20) were crossed to double transgenic Ptet-Cre/R26R reporter mice to generate TPH2-tTA/Ptet-Cre/R26R mice. Without Dox, 5-HT specific recombination started at E12.5. With permanent Dox administration, Ptet-controlled Cre-mediated recombination was absent. Dox withdrawal either postnatally or during adulthood induced efficient recombination in serotonergic neurons of all raphe nuclei, respectively. In the enteric nervous system, recombination could not be detected. We generated a transgenic mouse tTA line (TPH2-tTA) which allows both inducible and reversible transgene expression and inducible Cre-mediated gene deletion selectively in 5-HT neurons throughout life. This will allow precise delineation of serotonergic gene functions during development and adulthood.

## Introduction

Serotonin (5-HT) is the most mysterious of the main neuromodulators [1]. It appears to tune nearly all behaviours and physiological processes [2] and dysfunction of 5-HT neurons has been implicated in many neuropsychiatric disorders with diverse psychopathology [3]. Studies have indicated that the developmental timing of 5-HT disturbances together with gene-environment interactions is critical for observed changes in behaviour [4], [5], [6], [7], [8].

To delineate developmental and adult 5-HT specific gene effects on a wide spectrum of functions such as impulsivity/behavioural disinhibition, affective control, decision making or reinforcement learning, the ability for temporal and spatial control of gene manipulation in transgenic mouse models is a prerequisite. This is particularly important when timely manipulations of the 5-HT system are to be combined with specific environmental interventions such as maternal deprivation or housing isolation during development or adulthood.

Two widely used conditional transgenic systems allow for inducible, tissue-specific target gene manipulation, i.e. the tetracycline-inducible (tet-) system [9] and the CreERT/loxP-system [10]. In most cases, the CreERT/loxP-system is used for inducible gene knockouts although it also allows for inducible gene expression [11]. Vice versa, the tet-system is widely used for inducible and reversible expression of candidate genes albeit temporal control of tissue-specific gene deletion is also possible [12].

Inducible and reversible gene expression via the tet-system requires the generation of double-transgenic mice by mating a “transactivator line” with a “tet-response line”. In the transactivator line, a tissue specific promoter (TSP) controls the expression of an artificial transcription factor, the tetracycline transactivator (tTA or rtTA). In the tet-response line, a tet-promoter (Ptet) controls the transcription of a gene of interest (GOI). In double-transgenic TSP-tTA/Ptet-GOI mice, tissue-specific transactivator expression leads to Ptet-controlled transcription of the GOI which can be regulated by administration of doxycycline (Dox). The tet-off system, which was applied in this study, makes use of the tTA which can only bind and activate Ptet-controlled gene expression in the absence of Dox. In particular, the tet-system is suitable for reversible gene manipulations [13], which can not be accomplished with the Cre/loxP system where Cre-mediated recombination of loxP flanked sequences is irreversible. Finally, mating tissue-specific tTA mice with Ptet-Cre/loxP-flanked target gene mice allows spatially controlled, inducible gene deletion [12].

Here, we applied the tet-system to achieve inducible Ptet-controlled transgene expression specifically in 5-HT neurons. A 177 kb genomic *Tph2* sequence was chosen as an element to regulate tissue specific expression of tTA throughout life. Functional analysis of TPH2-tTA founder lines confirmed adult 5-HT neuron specific expression of the reporter gene lacZ in eight of eleven TPH2-tTA lines. TPH2-tTA founder L62-20 showed inducible and reversible Ptet-controlled β-galactosidase (βgal) expression in the majority of 5-HT neurons. Furthermore, Ptet-controlled Cre-mediated recombination allows efficient and temporally tightly controlled gene deletion as shown with triple-transgenic TPH2-tTA/Ptet-Cre/R26R mice. Hence, the TPH2-tTA line allows both inducible, 5-HT specific gene expression and gene knockout illustrating the versatility of this system to manipulate candidate genes bidirectionally.

## Results

### Generation of Transgenic Mice Expressing tTA in Serotonergic Neurons

**Figure 1:**
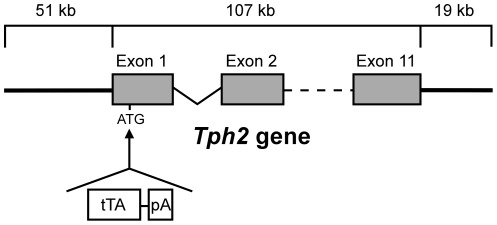
TPH2-tTA construct for DNA microinjection. A PCR-amplified tTA.pA sequence which contains flanking sequences (42 bp) homologous to the integration site of the PAC-based mouse *Tph2* gene was used for homologous recombination in E.coli. The construct was recombined into the ATG-start of Exon 1 of *Tph2*, thereby deleting a 23 bp sequence containing additional in-frame ATG sites.

Regulatory elements of the mouse *Tph2* locus were chosen to achieve spatial control of tTA expression in serotonergic neurons of the brain. We selected a PAC harbouring a 177 kb genomic insert containing the entire *Tph2* gene (107 kb) with additional 5′ upstream (51 kb) and 3′ downstream (19 kb) sequences. To generate the TPH2-tTA transgene the tTA coding sequence was inserted in place of the ATG-start of the Tph2 gene (Fig. 1) by homologous recombination in bacteria [14]. The modified genomic insert was introduced into the mouse germline of BDF1-mice (Charles River) by oocyte injections. 17 transgenic TPH2-tTA founders were identified by PCR-genotyping of tail DNA. Of those, 11 transmitted their transgene to the next generation and were further characterized.

### In situ Hybridization of tTA Expression in TPH2-tTA Founder Mice

**Figure 2:**
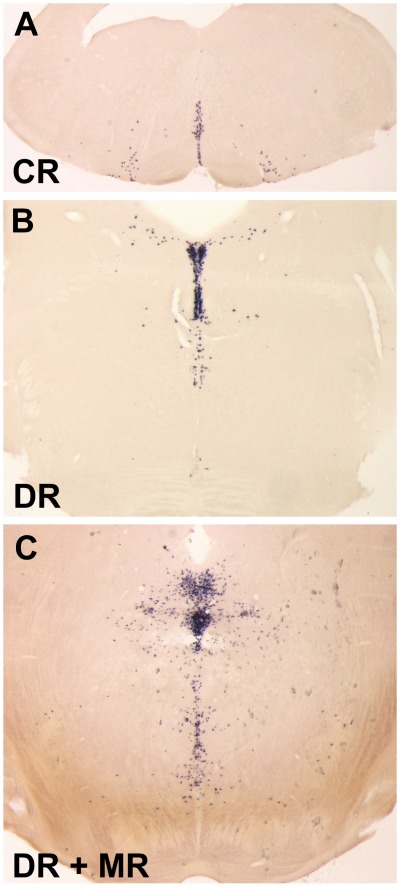
Non radioactive ***in-situ*** **hybridization of TPH2-tTA founder lines.** A tTA-antisense probe was used to detect tTA-mRNA in transgenic TPH2-tTA mice. Each TPH2-tTA line was screened by non-radioactive ISH. (A-C) Exemplary tTA *in situ* hybridization of founder line 62-20 showed abundant tTA-mRNA in brain stem and midbrain areas where raphe nuclei with serotonergic neurons are located. CR, caudal raphe nuclei; DR, dorsal raphe nuclei; MR, median raphe nuclei.

**Figure 3:**
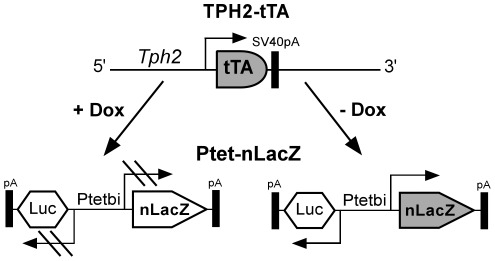
Functional characterization of Ptet-controlled βgal expression in TPH2-tTA/Ptet-nLacZ mice. TPH2-tTA mice were mated with Ptet-nLacZ mice to generate double-transgenic TPH2-tTA/Ptet-nLacZ mice. In Ptet-nLacZ reporter mice, a bidirectional promoter Ptetbi which contains seven tet operator sequences flanked by two minimal promoters [77] allows bidirectional Ptet-controlled transcription of nuclear localized lacZ and luciferase cDNA. Only βgal reporter expression was analyzed in TPH2-tTA/Ptet-nLacZ mice. In the presence of Dox (+Dox), βgal expression is not initiated since tTA binding to Ptet is prevented by Dox. In the absence of Dox (-Dox), tTA binds to Ptet which activates lacZ reporter gene transcription.

First, we investigated tTA mRNA expression by non-radioactive in situ-hybridization (ISH) on coronal vibratome sections of the brain using a specific antisense tTA probe. Nine of eleven TPH2-tTA lines showed tTA mRNA expression in the brain stem and midbrain where serotonergic neurons are located (Fig. 2). All tTA-mRNA positive TPH2-tTA lines were crossed with Ptet-nLacZ reporter mice [15] to functionally assess Ptet-regulated reporter gene expression (Fig. 3).

### Functional Characterization of Adult TPH2-tTA Founder Mice

**Figure 4:**
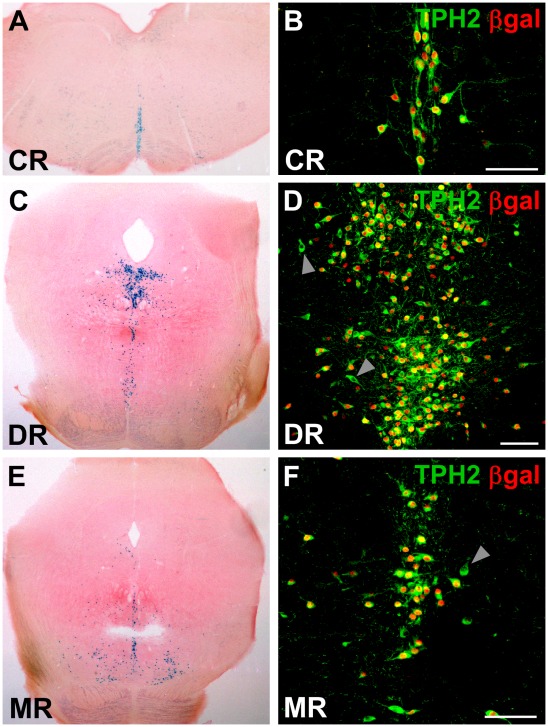
5-HT neuron-specific transgene expression in TPH2-tTA/Ptet-nLacZ mice. Exemplary results from TPH2-tTA line L62-20 mated with Ptet-nLacZ mice to generate double-transgenic TPH2-tTA/Ptet-nLacZ mice which never received Dox. Adult mice (P90) were sacrificed and analyzed with X-Gal staining and IHC. (A,C,E) Without Dox administration, X-Gal staining of coronal sections of brain stem and midbrain showed intense βgal activity in the raphe nuclei where 5-HT neurons reside. (B,D,F) Dual-label fluorescence IHC with βgal and 5-HT neuron-specific TPH2 antibodies. Co-labelling indicates 5-HT neuron-specific βgal transgene expression. IHC confirmed that βgal expression occurred tissue-specifically in the majority of all 5-HT neurons (B,D,F). Grey arrowheads show scarce βgal-negative TPH2+5-HT neurons. Scale bars: 100 µm.

Initial functional characterization of all TPH2-tTA lines was conducted without Dox to keep the tet-system permanently active (Fig. 3). Adult TPH2-tTA/Ptet-nLacZ mice, 2–3 months of age, were sacrificed for analysis. In all founder lines, X-Gal staining of TPH2-tTA/Ptet-nLacZ mice detected βgal activity in the brain stem and mid brain which are the neuroanatomical areas where serotonergic neurons are located (Fig. 4A,C,E). Dual-label fluorescence immunohistochemistry (IHC) with βgal and tryptophan hydroxylase 2 (TPH2) antibodies was performed to investigate tissue-specificity and expression levels of Ptet-controlled βgal in serotonergic neurons. While βgal expression confirms successful tTA-induction of Ptet-controlled lacZ transcription, TPH2 antibodies detect the tryptophan hydroxylase 2 (TPH2) which is the rate-limiting enzyme of brain 5-HT synthesis and consistently found in all serotonergic neurons of the raphe nuclei [16]. Dual-label fluorescence IHC confirmed that Ptet-controlled βgal expression occurred selectively in 5-HT neurons (Fig. 4B,D,F).

With the exception of line L62-31 which showed aberrant expression outside the raphe nuclei, all other founder lines were negative for X-Gal staining in extraserotonergic brain regions. Nonetheless, the amount of βgal expression differed significantly among TPH2-tTA founder lines and even among TPH2-tTA/Ptet-nLacZ offspring from the same TPH2-tTA line.

As a consequence, we decided to extensively study the eight remaining 5-HT neuron specific TPH2-tTA founder lines. Since developmental Ptet-controlled transgene suppression in the presence of Dox is a desired feature in the tet-off system, we performed all further analysis with Dox suppression during development until P60. Hereafter, Dox was withdrawn to induce Ptet-controlled transgene expression and mice were sacrificed for analysis at P90. Each TPH2-tTA founder line was crossed to homozygous Ptet-nLacZ mice to generate large numbers of TPH2-tTA/Ptet-nLacZ offspring. Adult offspring from 2–3 different matings (TPH2-tTA×Ptet-nLacZ) were analyzed individually for βgal expression (n  = 5–14 mice for each line). Every TPH2-tTA founder line showed tissue-specific βgal expression in serotonergic neurons (Table 1). Nonetheless, in several TPH2-tTA lines (L62-22, L62-26, L62-29, L62-30, L62-34) we found highly variable, serotonergic βgal expression among offspring. This is an undesired feature as this would lead to highly unpredictable candidate gene expression in individual mice during experiments. In contrast, in the three remaining founder lines (L62-1, L62-20, L62-25) the level of 5-HT neuron specific βgal expression among TPH2-tTA/Ptet-nLacZ offspring was relatively stable while the absolute efficiency of βgal expression in 5-HT neurons (βgal+/TPH2+ of all TPH2+ neurons) varied significantly among these lines (Table 1). Line 62-20 displayed the highest level of overall βgal expression in TPH2+ serotonergic neurons (81% of all 5-HT neurons, 95%-CI: 80–82%) together with low variability of βgal expression among double-transgenic offspring (Table 1). When subdivided, 93%, 81% and 71% of all TPH2+ neurons were βgal+ in the caudal, dorsal and median raphe nuclei, respectively (95%-Confidence interval (CI): caudal 90–95%, dorsal 80–83%, median 67–74%) (Table 1). Based on these findings, TPH2-tTA line 62-20 was selected for all further experiments.

**Table 1:** Efficiency of Ptet-controlled βgal expression in TPH2-tTA/Ptet-nLacZ offspring of eight independent TPH2-tTA founder lines.

Line	No. of analyze offspring	absolute frequency(βgal+/TPH2+):TPH2+	relative frequency(βgal+/TPH2+):TPH2+	range of relative frequency	95% CI
L62-1 CR		268∶407	66%	52–78%	61–70%
L62-1 DR		1161∶2596	45%	37–50%	43–47%
L62-1 MR		152∶483	31%	18–54%	27–36%
L62-1 total	7	1581∶3486	45%	38–53%	44–47%
L62-20 CR		494∶533	93%	89–96%	90–95%
L62-20 DR		2587∶3186	81%	72–89%	80–83%
L62-20 MR		486∶686	71%	63–86%	67–74%
L62-20 total	9	3567∶4405	81%	74–89%	80–82%
L62-22 CR		376∶421	89%	70–100%	86–92%
L62-22 DR		1353∶2081	65%	40–83%	63–67%
L62-22 MR		277∶535	52%	30–65%	47–56%
L62-22 total	10	2006∶3037	66%	42–81%	64–68%
L62-25 CR		79∶247	32%	24–38%	26–38%
L62-25 DR		152∶1114	14%	5–19%	12–16%
L62-25 MR		53∶278	19%	6–33%	15–24%
L62-25 total	5	284∶1639	17%	9–23%	16–19%
L62-26 CR		418∶467	90%	55–100%	86–92%
L62-26 DR		2765∶3419	81%	76–91%	80–82%
L62-26 MR		330∶522	63%	36–72%	59–67%
L62-26 total	9	3513∶4408	80%	76–85%	79–81%
L62-29 CR		316∶512	62%	25–89%	57–66%
L62-29 DR		1162∶3493	33%	18–51%	32–35%
L62-29 MR		245∶731	34%	19–49%	30–37%
L62-29 total	9	1723∶4736	36%	19–53%	35–38%
L62-30 CR		636∶742	86%	51–99%	83–88%
L62-30 DR		3470∶4266	81%	57–94%	80–83%
L62-30 MR		475∶774	61%	35–84%	58–65%
L62-30 total	12	4581∶5782	79%	54–89%	78–80%
L62-34 CR		399∶830	48%	6–86%	45–52%
L62-34 DR		2456∶5332	46%	3–69%	45–47%
L62-34 MR		404∶1124	36%	8–69%	33–39%
L62-34 total	14	3259∶7286	45%	4–66%	44–46%

TPH2-tTA/Ptet-nLacZ nice which had received Dox throughout development until P60 were sacrificed at P90 after 30 days of Ptet-controlled gene activation without Dox and analyzed with dual-label IHC. Efficiencies for caudal (CR), median (MR) and dorsal (DR) raphe nuclei were separately and jointly (total) calculated. Confidence-bounds (CI) were calculated using the Clopper-Pearson method based on significance level 95.0%.

### Inducible and Reversible Tissue Specific βgal Expression in TPH2-tTA/Ptet-nLacZ Mice

**Figure 5:**
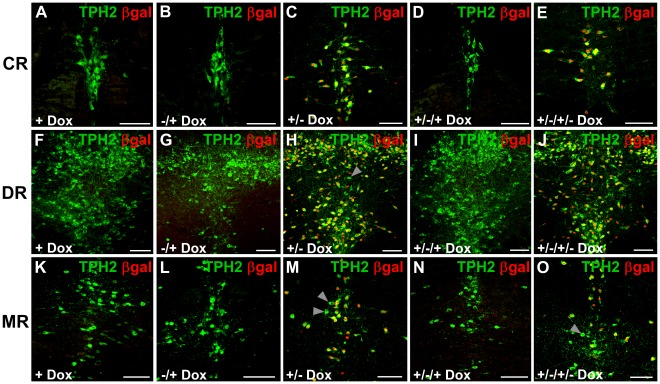
Inducible and reversible 5-HT neuron-specific transgene expression in TPH2-tTA/Ptet-nLacZ mice. Inducibility and reversibility of Ptet-controlled transgene expression is a hallmark of the tet-system. To test these potential merits in TPH2-tTA mice, double-transgenic TPH2-tTA/Ptet-nLacZ mice were generated. Adult mice (>P90) were sacrificed and analyzed with dual-label fluorescence IHC using βgal and 5-HT neuron-specific TPH2 antibodies. (A,F,K) With permanent Dox administration (+Dox), Ptet-controlled βgal expression could be fully suppressed in TPH2-tTA/Ptet-nLacZ mice. Furthermore, Ptet-controlled βgal expression was reversible with Dox administration, regardless whether prior βgal expression occurred during development (−/+Dox; B,G,L) or during adulthood (+/−/+Dox; D,I,N). Vice versa, after chronic Dox administration during embryonic and postnatal development, Ptet-controlled βgal expression could be fully induced (C,H,M) and re-induced (E,J,O) via Dox withdrawal. Scale bars: 100 µm.

The tet-system allows for inducible and reversible gene expression (Fig. 3) which is a unique hallmark of this system and clearly distinguishes it from other conditional systems based on recombinases (Cre, Flp). Hence, the TPH2-tTA line L62-20 was further tested for inducibility and reversibility of Ptet-controlled reporter gene expression (Table 2; Fig. 5). Using the tet-off system, inducible gene expression requires permanent Dox treatment to suppress gene expression prior to induction/activation without Dox (+/−Dox). In contrast, reversible gene expression requires a previously activated system to be shut down with Dox (−/+Dox). While suppression of Ptet-controlled gene expression with Dox is rapidly achieved using the tet-system, activation of gene expression after Dox withdrawal may require up to several weeks. After permanent Dox administration until P60, partial activation of Ptet-controlled gene expression in TPH2-tTA/Ptet-nLacZ mice was detectable after 7 days whereas maximal Ptet-controlled gene expression was achieved after 3 weeks (data not shown). As a consequence, in the subsequent analysis activation and reactivation was measured 30 days after Dox withdrawal.

**Table 2:** Protocols for inducible and reversible reporter gene expression in TPH2-tTA/Ptet-nLacZ mice.

Protocol 1:	Protocol 2:	Protocol 3:	Protocol 4:	Protocol 5:
+Dox	θ/+Dox	+/θ Dox	+/θ/+ Dox	+//+/θ Dox
+Dox untilP90	θDox untilP60	+Dox untilP60	+Dox untilP60	+Dox untilP60
	+Dox untilP90	θDox untilP90	θDox untilP90	θDox untilP90
			+Dox until P120	+Dox untilP120
				θDox untilP150

Dox was administered with 5 µg/ml from initiation of matings until birth. At P0, Dox concentration was changed to 50 µg/ml. Suppression of Ptet-controlled gene expression in adult mice was also done with 50 µg/ml Dox. In protocol 1, mice were maintained under chronic Dox administration throughout life until sacrifice at P90. In protocol 2, transgenic mice were maintained in the absence of Dox until P60 when Dox administration was initiated until sacrifice at P90. In protocol 3, Dox was given from conception until P60 when Dox was withdrawn until sacrifice at P90. Protocol 4 is based on protocol 3. Here, Ptet-controlled βgal expression was re-suppressed from P90 until sacrifice at P120. Protocol 5 is based on protocol 4, but removal of Dox at P120 until sacrifice at P150 reactivated Ptet-controlled βgal expression. +Dox, administration of Doxycycline; θDox, no administration of Dox.

To analyze inducible and reversible gene expression in detail, the first group of double-transgenic mice was given Dox permanently during development and early adulthood until sacrifice at P90 (+Dox) to analyze chronic suppression of Ptet-regulated gene expression (Table 2: protocol 1; Fig. 5A,F,K) In protocol 2, mice which had not received Dox during development until P60 were given Dox until sacrifice at P90 (−/+Dox) to analyze reversibility of previously activated gene expression n during development (Table 2: protocol 2; Fig. 5B,G,L). In protocol 3, mice were given Dox during embryonic and postnatal development until P60 when Dox was withdrawn until sacrifice at P90 (+/−Dox) to test for inducible Ptet-controlled gene activation during adulthood (Table 2: protocol 3; Fig. 5C,H,M). In protocol 4, mice received the same protocol as in 3 but were given Dox again from P90 until sacrifice at P120 (+/−/+ Dox) to analyze for reversibility of induced Ptet-controlled gene expression during adulthood (Table 2: protocol 4; Fig. 5D,I,N). In protocol 5, mice received the same protocol as in 4 but Dox was withdrawn again from P120 until sacrifice at P150 (+/−/+/− Dox) to test for re-activation of Ptet-controlled gene expression (Table 2: protocol 5; Fig. 5E,J,O). All mice were sacrificed at the respective points in time and brain sections were analyzed with X-Gal staining (X-Gal) and dual-label immunohistochemistry (IHC) for Ptet-controlled reporter gene (βgal) expression.

A prerequisite for inducible gene expression with the tet-off system is its suppressibility with Dox. We show that Ptet-controlled serotonergic gene expression could be fully suppressed with chronic Dox administration (Table 2: protocol 1, Fig. 5A,F,K). Equally important, under all tested conditions, βgal expression could be completely reversed with Dox administration (Table 2: protocol 2 and 4), irrespective of previous activation during development (Table 2: protocol 2; Fig. 5B,G,L) or adulthood (Table 2: protocol 4; Fig. 5D,I,N). Vice versa, induction of Ptet-controlled gene expression was always feasible, regardless whether gene expression was previously suppressed with Dox during development (Table 2: protocol 3; Fig. 5C,H,M) or adulthood (Table 2: protocol 5; Fig. 5E,J,O). Thus, Ptet-controlled gene expression in the 5-HT neuronal system can be manipulated at will in all possible combinations, as gene expression can be repeatedly induced and/or reversed at any time during the animal’s life.

### Tight Control of Tissue-specific Recombination in TPH2-tTA/Ptet-Cre/R26R Mice During Development and Adulthood

**Figure 6:**
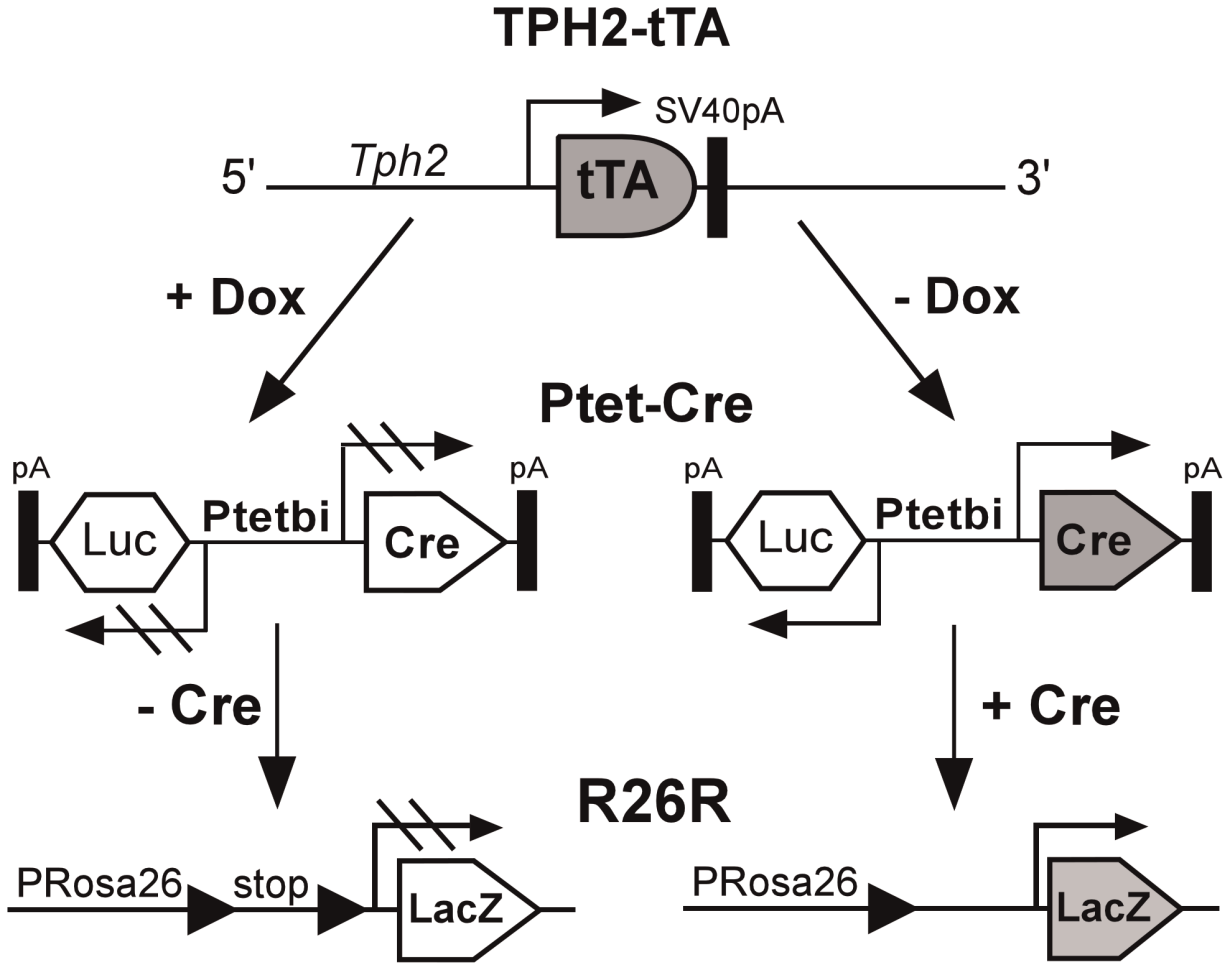
Functional characterization of Ptet-controlled, Cre-mediated recombination in TPH2-tTA/Ptet-Cre/R26R mice. TPH2-tTA mice were mated with Ptet-Cre/R26R mice to generate triple-transgenic TPH2-tTA/Ptet-Cre/R26R mice. In Ptet-Cre mice, a bidirectional promoter Ptetbi allows co-regulated Ptet-controlled transcription of Cre and luciferase cDNA. Luciferase activity can be used to indirectly quantify tissue-specific Cre expression but was not needed to assess Cre-mediated recombination. In the presence of Dox (+Dox), Ptet-controlled Cre expression does not occur. In the absence of Dox (−Dox), tTA activates Ptet-controlled Cre expression. Cre then deletes a loxP-flanked stop cassette in the Rosa26 locus, thereby initiating βgal expression which indicates successful Cre-mediated recombination.

Heretofore, we showed that TPH2-tTA mice permit tissue-specific, inducible and reversible Ptet-controlled gene expression in serotonergic neurons. Next, we asked whether TPH2-tTA mice also allow for inducible gene deletion in combination with the Cre/loxP system (Table 3). For this purpose, we generated triple-transgenic TPH2-tTA/Ptet-Cre/R26R mice (Fig. 6). In these mice, binding of tTA to the Ptet-promoter in the absence of Dox induces Cre expression (Ptet-Cre) [12]. This in turn leads to Cre mediated excision of a loxP-flanked stop-cassette in the Rosa26 locus which results in βgal expression (R26R) [17]. Hence, βgal expression functionally confirms tTA-induced, Ptet-controlled Cre/loxP recombination.

**Table 3:** Protocols for inducible recombination in TPH2-tTA/Ptet-Cre/R26R mice.

Protocol 1:	Protocol 2:	Protocol 3:	Protocol 4:
θDox	+Dox	+/θDox postnatal	+/θ Dox adult
θDox until P90	+Dox until P90	+Dox until P0	+Dox until P60
		θDox until P90	θDox until P90

Dox was administered with 5 µg/ml from initiation of matings until birth. At P0, Dox concentration was changed to 50 µg/ml. In protocol 1, transgenic mice were maintained in the absence of Dox throughout life until sacrifice at P90. In protocol 2, mice were maintained under chronic Dox administration throughout life until sacrifice at P90. In protocol 3, Dox was given from conception until P0 when it was withdrawn until sacrifice at P90. In protocol 4, Dox was given from conception until P60 when Dox was withdrawn until sacrifice at P90. +Dox, administration of Doxycycline; θDox, no administration of Dox.

**Figure 7:**
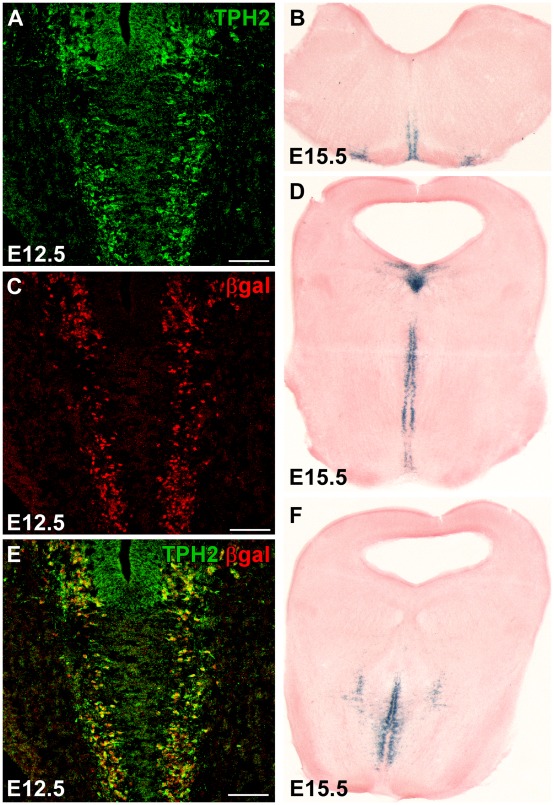
Embryonic serotonergic recombination in TPH2-tTA/Ptet-Cre/R26R mice. In the absence of Dox, Ptet-controlled, Cre-mediated recombination was analyzed in the embryonic brain. Double-fluorescence IHC with βgal- and 5-HT neuron-specific TPH2 antibodies was done in brains from embryos at E12.5–E15.5. Already at E12.5, extensive βgal-staining was observed in developing 5-HT neurons (A,C,E). Later during development at E15.5, X-Gal staining allows easy visualization of the strong and ubiquitous βgal activity in caudal (B), dorsal (D) and median (D,F) raphe nuclei.

**Figure 8:**
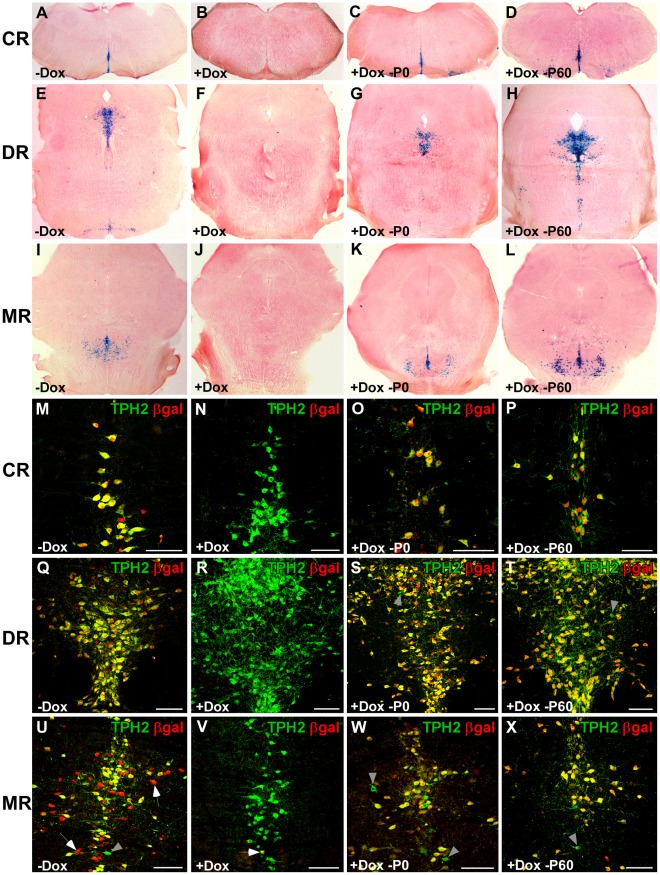
Inducible, 5-HT neuron-specific recombination in TPH2-tTA/Ptet-Cre/R26R mice. Adult mice (P90) were sacrificed and analyzed with X-Gal staining and IHC. (A–L) X-Gal staining of coronal sections of the brain stem and midbrain. (M–X) Dual-label fluorescence IHC with βgal and 5-HT neuron-specific TPH2 antibodies. Co-labelling indicates 5-HT neuron-specific recombination. Without Dox administration (−Dox), X-Gal staining showed intense βgal activity in all areas of the raphe nuclei where 5-HT neurons are located (A,E,I). Dual-label IHC confirmed that βgal expression occurs tissue-specifically in virtually all 5-HT neurons (M,Q,U). Of note, extraserotonergic βgal expression was found in the median raphe nuclei of untreated mice (U, arrows). (B,F,J,N,R,V) With permanent Dox administration (+Dox), Cre-induced recombination and consequently βgal expression did only occur in a minor fraction of 5-HT neurons of TPH2-tTA/Ptet-Cre/R26R mice (V, arrow). To assess inducibility of Cre-mediated recombination, Dox was either administered during embryonic development until P0 (+Dox –P0; C,G,K,O,S,W) or during embryonic and postnatal development until P60 (+Dox –P60; D,H,L,P,T,X), when Dox was withdrawn until sacrifice at P90. X-Gal staining showed equally strong βgal activity in the raphe nuclei of TPH2-tTA/Ptet-Cre/R26R mice which were induced at P0 (C,G,K) or at P60 (D,H,L). As shown with IHC, recombination occurred tissue-specifically in the majority of 5-HT neurons, irrespective of the time point of induction at P0 (O,S,W) or P60 (P,T,X). The efficiency of tissue-specific recombination was indistinguishable from mice which never received Dox (M,Q,U) which proves that induction of Cre-mediated recombination after Dox suppression is equally efficient. Of note, when Cre recombination was suppressed with Dox until P0, extraserotonergic recombination in the median raphe nuclei could not be detected anymore (W,X). Grey arrowheads indicate scarce non-recombined, βgal-negative TPH2+5-HT neurons. Scale bars: 100 µm.

First, TPH2-tTA/Ptet-Cre/R26R mice which had never received Dox were sacrificed between E12.5 and E15.5 to evaluate whether the transgenic TPH2 regulatory elements adequately control tTA expression early during development of 5-HT neurons. At E12.5, TPH2-tTA/Ptet-Cre/R26R mice already show extensive βgal expression in serotonergic neurons (Fig. 7A,C,E), one day after TPH2 protein expression in 5-HT neurons begins [18]. Hence, Ptet-controlled gene expression during embryonic development is adequately regulated in TPH2-tTA mice. Consequently, adult TPH2-tTA/Ptet-Cre/R26R mice which had never received Dox also showed extensive X-Gal staining in the raphe nuclei (Table 3: protocol 1; Fig. 8A,E,I). Using dual-label fluorescence IHC against TPH2 and βgal, massive recombination, i.e. βgal expression, in 5-HT neurons of all raphe nuclei of the brain stem and midbrain during development could be confirmed (Fig. 8M,Q,U). Extraserotonergic brain regions were devoid of βgal activity except for scarce staining in the hypothalamus and beneath the aqueduct. Of note, single βgal+/TPH2-negative cells were identified within the median raphe nuclei of TPH2-tTA/Ptet-Cre/R26R mice which had never received Dox (Fig. 8U). With permanent Dox administration (Table 3: protocol 2), recombination could not be detected with X-Gal staining and dual-label IHC in TPH2-tTA/Ptet-Cre/R26R mice at P90 (Fig. 8B,F,J,N,R,V). Hence, Dox application allows permanent and complete suppression of Ptet-controlled Cre-mediated recombination throughout development and adulthood.

Next, we assessed inducible activation of Ptet-controlled, Cre-mediated gene deletion in 5-HT neurons (Table 3: protocol 3 and protocol 4). When Dox was given throughout embryogenesis until P0 and mice were sacrificed at P90, virtually all 5-HT neurons in adult mice were βgal+, indicating efficient, inducible postnatal recombination in all raphe nuclei (Table 3: protocol 3; Fig. 8O,S,W). Interestingly, Dox suppression during embryogenesis abolished recombination in TPH2-negative cells in the median raphe nuclei (Fig. 8W). Furthermore, the minimal extraserotonergic recombination in the hypothalamus and beneath the aqueduct was not observed any longer. When Dox was given continuously until P60 and mice were again sacrificed at P90, X-Gal staining revealed extensive staining in the brain stem and mid brain where serotonergic neurons are located (Table 3: protocol 4; Fig. 8D,H,L). Dual-label IHC confirmed that the majority of 5-HT neurons (TPH2+) showed recombination by βgal expression (>90%) while βgal expression outside the raphe nuclei was absent confirming serotonergic tissue-specificity (Fig. 8P,T,X).

Previously, TPH2 expression was reported in the enteric nervous system [18], [19]. We used TPH2-tTA/Ptet-Cre/R26R mice (P21) which had never received Dox to determine βgal expression in the gut by IHC. On coronal sections throughout the gastrointestinal system we could not detect βgal staining which suggests that transgenic TPH2-tTA mice do not express the transactivator in enteric neurons.

## Discussion

In this study, we describe a TPH2-tTA mouse line which allows efficient, Dox-repressible, Ptet-controlled gene expression in serotonergic neurons of the brain. We demonstrate the functionality and feasibility of our approach by generating transgenic TPH2-tTA/reporter mice which either show activatable and reversible 5-HT neuron specific gene overexpression or inducible Cre-mediated gene deletion at any time during development and adulthood.

### Transgenic 5-HT Neuron-specific Regulation of Candidate Genes

For the 5-HT system, recent advances have led to the identification of tissue-specific promoter sequences which allow Cre and CreERT2 expression exclusively in serotonergic neurons [20], [21], [22], [23], [24]. 5-HT specific gene deletion has led to a wealth of new findings regarding the development and functioning of the 5-HT system in physiological and pathophysiological processes [20], [25], [26], [27], [28], [29]. However, for the better understanding of the diverse functions of the 5-HT system, it will not only be of interest to delete genes, but also to enhance the expression of certain genes or even to induce serotonergic misexpression. To achieve this task, the most applied gene regulatory system in transgenic animals is the tet-system [30]. Tissue-specific promoters have been successfully employed to spatially control transactivator expression in the brain of transgenic mice [31], [32], [33], [34], [35], [36], [37], [38] but a 5-HT neuron specific transactivator line has not been published to date.

Expression of TPH2 begins at E11 and persists throughout life [18]. Its expression is restricted to serotonergic raphe nuclei in the mid brain and brain stem [39]. In this study we implemented regulatory sequences of the brain specific Tph2 gene [39] to ensure cell-type specific serotonergic tTA expression. The 177 kb PAC-derived DNA fragment containing the entire mouse *Tph2* gene plus additional large flanking regions reliably directed tTA expression solely to serotonergic neurons in postnatal and adult mice. The same *Tph2* DNA sequence was previously shown to adequately control CreERT2 expression in serotonergic neurons of transgenic TPH2-CreERT2 mice and rats [21], [40]. During embryonic development, we found transgenic expression in a small number of TPH2-negative cells of the median raphe nuclei when TPH2-tTA mice (-Dox) or TPH2-CreERT2 mice (+ tamoxifen E15–18) [21] were studied. This may indicate aberrant embryonic tTA- or CreERT2 expression due to missing regulatory elements of the TPH2-construct specific for the embryonic phase. Alternatively, the close anatomical relationship of βgal+/TPH2-negative cells with βgal+/TPH2+5-HT neurons may indicate that there is a transient embryonic TPH2 expression in non-serotonergic cells in the raphe nuclei or that some originally serotonergic cells convert to non-serotonergic neurons.

### The TPH2-tTA Line 62-20 Allows Inducible and Reversible Ptet-controlled Gene Expression

The generation of transgenic transactivator and tet-response lines is sensitive to various factors influencing its regulatory potential [41], [42], [43]. This comes as no surprise as the regulatory magnificence, allowing for activatable and reversible gene expression, relies heavily on its permanent controllability. Compared to the Cre/loxP system which induces an irreversible process of gene activation or gene deletion by excising loxP-flanked DNA sequences, the tet-system requires permanent tTA activation of the tet-promoter to transcribe the Ptet-controlled transgene. This process requires sufficient levels of tissue-specific tTA expression and constant accessibility of the Ptet-transgenic locus, also after prolonged periods of silencing with Dox.

Both the transactivator and the Ptet transgene are sensitive to genomic position integration effects, in particular if small plasmid-based DNA sequences are microinjected [42]. This holds true for any randomly integrated transgene but is particularly worrisome in an inducible system which requires two transgenes to optimally work together. Position effects can be minimized by using large genomic sequences to drive transactivator or Ptet-controlled gene expression [42], [44]. For that reason, we used a large genomic Tph2 sequence to insulate tTA expression from position effects. As expected, this strategy ensured faithful tissue-specific serotonergic expression in 8 of 11 lines.

Variable Ptet-controlled gene expression has been observed in sibling mice carrying identical transgenes inherited from the same parents [42], [45], [46]. Similar gene expression variegation was also found in most of our TPH2-tTA founder lines. Extensive expression screening of double-transgenic TPH2-tTA/Ptet-nLacZ litters from all functional TPH2-tTA lines identified one TPH2-tTA line (L62-20) which showed robust Ptet-controlled transgene expression. Apart from efficient reporter gene expression in more than 80% of 5-HT neurons, expression variability among double-transgenic offspring was very low and Ptet-controlled gene expression could be turned on and off at will.

Hence, the characteristics of the TPH2-tTA line 62-20 allow for reliable temporal control of candidate gene expression in the majority of 5-HT neurons. Developmental suppression of Ptet-controlled candidate gene expression and adult activation can be important if one wishes to exclusively study the adult function of a particular gene of interest and wishes to exclude developmental effects of transgene expression. Indeed, overexpressing a protein that serves essential functions during embryogenesis can result in early lethality. Many genes also exert multiple functions in distinct cell types during ontogeny and postnatally (pleiotropy). This may result in complex phenotypes and, thereby, in difficulties in distinguishing cell-autonomous from more complex origins of abnormalities [47]. Finally, developmental transgene overexpression may also lead to adaptive changes during development. This will either lead to the disappearance of an abnormal phenotype if homeostasis has been achieved or to a phenotype that is not directly related to the overexpressed gene but to the adaptive changes caused by the manipulated gene. On the other hand, there are scientific questions which are best answered with a system that allows reversibility of gene expression. While the CreERT2/loxP system allows for temporal control of transgene expression via tamoxifen treatment, these changes are irreversible compared to transgenic expression using the tet-system. Shutting down gene expression allows to assess whether induced phenotypic changes are reversible or whether they persist independent of the initial genetic manipulation [48], [49], [50], [51]. Of note, while Ptet-controlled gene expression can be rapidly suppressed with Dox within 1–2 days during embryonic [52], [53] and postnatal [36] development as well as during adulthood [42], [54], gene activation after Dox withdrawal cannot be initiated with the same speed at any stage during life [36], [37], [42], [54]. Variables which influence the delay phase for activation of Ptet-controlled gene expression include the choice of doxycycline or tetracycline for gene suppression [42], duration of Dox treatment [36], [51], Dox dosage [37], [42] and tissue involved [55], [56]. Studies which used Dox concentrations between 50-2000 µg/ml found that earliest Ptet-controlled gene expression commenced around 2–5 days after Dox withdrawal with full activation of gene expression between 7 days and 8 weeks [36], [37], [42], [57]. Hence, the delay phase of Ptet-controlled gene activation after Dox treatment is highly variable and appears to be mostly dependent on the duration of Dox treatment and its dosage [37], [42], [58]. In double-transgenic TPH2-tTA/Ptet-nLacZ and triple-transgenic TPH2-tTA/Ptet-Cre/R26R mice which were suppressed with 5 µg/ml Dox (to the mothers) from conception until birth followed by 50 µg/ml until P60, we found partial activation of βgal expression after 7 days and full activation after 3–4 weeks. Thus, the Dox withdrawal time point is not equivalent to the initiation of effective Ptet-controlled gene expression. This delay phase needs to be empirically determined if Ptet-controlled gene expression in TPH2-tTA mice is intended to be initiated at a certain point in time during development or adulthood.

Another concern of prolonged Dox administration during embryonic development is the deposition of Dox in calcified tissues of the embryo which is associated with bone growth retardation [59]. Recently, a delay in bone growth has been shown in rats treated with 8 mg/kg Dox from E8–19 [60]. We and many others have never found growth deficits in mice treated with Dox but can not rule out that a delay of skeletal differentiation occurs during development. It has been reported that low dosages of Dox do not result in clinically relevant blood levels and are thus unlikely to produce deleterious effects [37]. During embryonic development, we administer only 5 µg/ml Dox to pregnant mothers which is to our knowledge the lowest Dox concentration so far applied which sufficiently suppresses Ptet-controlled gene expression *in vivo*. Hence, pregnant TPH2-tTA mice and their fetuses receive only 0.75–1.25 mg/kg/d Dox which is an approximately 10 times lower Dox dosage than previously reported [60].

### TPH2-tTA Mice Allow Inducible Ptet-controlled, Cre-mediated Gene Deletion

The tet-technology can be combined with the Cre/loxP system. For this purpose, a tissue-specific transactivator line has to be bred to a Cre line in which Cre expression is controlled by a tet-promoter (Ptet-Cre). A powerful tool for this approach is the well characterized LC-1 mouse line, where Ptet-controlled Cre synthesis is tightly controlled yet highly inducible [12]. In many aspects, this approach compares favorably to the widely used tamoxifen-inducible CreERT2 system. Tamoxifen is a partial estrogen/antiestrogen in humans and a full estrogen in mice [61]. In human and rodents, acute and late side effects (http://www.nlm.nih.gov/medlineplus/druginfo/meds/a682414.html) have been documented for this substance [62], [63], so its application cannot be presumed to be inert for transgenic mice. Hence, molecular and cellular processes, in particular neuroendocrine pathways, will be altered with tamoxifen administration.

In most studies, tamoxifen is administered via repeated intraperitoneal (ip) injections or oral gavages which are both stressful procedures for the mice. Regardless of the administration route, tamoxifen administration during embryonic development is problematic. It has been reported to cause spontaneous abortion and intrauterine hemorrhage as well as developmental delay and abnormal head development in the embryos [64], [65]. It also inhibits natural birth requiring standard caesarian section and foster mothers for the pups of tamoxifen treated pregnant mice [21], [66], [67]. Postnatally, the route of administration usually requires repeated intraperitoneal injections or oral gavages to the lactating mothers, which imposes significant stress to the mothers and pups. While others have reported on successful repeated intraperitoneal injections or oral gavages to lactating mothers [64], [68], in our hands this procedure has led to frequent maternal abandonment of the pups. Although it has been shown that adult tamoxifen administration had no effect on subsequent testing of several behavioral parameters [69], it has not been clarified whether this is also true for tamoxifen application during development. Only recently, several studies reported on successful recombination after administration of tamoxifen citrate via mouse chow [70], [71], [72], [73].

A sensible alternative to the inducible CreERT2 system to circumvent tamoxifen side effects and stressful administration routes is the generation of tissue-specific transactivator/Ptet-Cre mice which allows inducible overexpression or gene deletion without tamoxifen administration. In this study, we confirm the feasibility of this approach by generating TPH2-tTA/Ptet-Cre/R26R mice. Ptet-controlled Cre expression could be successfully suppressed with oral Dox administration via drinking water at any time during development and adulthood. Controlling Cre expression limits recombinase activity to a defined time window thereby avoiding Cre toxicity which occurs during prolonged Cre expression [74]. Removing Dox from the drinking water resulted in successful embryonic, postnatal or adulthood deletion of loxP flanked DNA sequences allowing for an easy, non-interventional, non-toxic, temporal control of gene deletion.

Transgenic TPH2-tTA mice provide an opportunity to study highly efficient, inducible and reversible expression of Ptet-controlled transgenes in 5-HT neurons. Moreover, in combination with a Ptet-inducible Cre line we demonstrate an “all in one tool” which also allows Cre-mediated gene deletion selectively in serotonergic neurons without any background recombination. The option to inducibly express or delete the same candidate gene diametrically at any time during life will promote the understanding of molecular, biochemical and behavioural effects of time-specific gene manipulations within the serotonergic system.

## Materials and Methods

### Generation of Transgenic TPH2-tTA Mice

A modified 196 kb PAC [21] that contains the full-length mouse Tph2 gene (107 kb) and 51 kb upstream and 19 kb downstream DNA sequences was selected for recombineering in EL250 bacteria [14]. The stTA2 sequence was PCR amplified together with the SV40 polyadenylation (polyA) site from the plasmid pUHT 61-1 [75] and ligated into the pL451 vector [14] harbouring a FRT flanked Neomycin/Kanamycin (Neo/Kan) resistance cassette. The resulting stTA2.SV40polyA-FRT.Neo/Kan.FRT-sequence was PCR-amplified using primers that comprised 42 bp of DNA-sequence homologous to the selected recombination sites in Exon 1 of the *Tph2* gene (upstream primer 5′-TGGTCCCCCCTGCTGCTGAGAAAGAAAATTACATCGGGAGCCatgtctagactggacaagag caaag-3′, downstream primer 5′-GAATCCAAGGACAACCCTCTCCTGGCCCAGTATTTACTGGAAatattatgtacctgactgatg aag-3′). This DNA fragment was used for homologous recombination of the tTA.polyA sequence into the *Tph2* start codon of the 196 kb PAC. A 23 bp sequence starting with the ATG-translation start of *Tph2* was intentionally deleted since it contained additional in-frame ATG-start sites. The PCR-product (200 ng) was electroporated into EL250 bacterial cells containing the Chloramphenicol-resistent pPAC4-L065 (pPAC4-Cam-L065) and homologous recombination was heat-induced. Next, Flp-mediated excision of the FRT-flanked Kanamycin-resistence gene was achieved by Arabinose induction. Finally, recombination of stTA2.pA into the ATG-start of Exon 1 was verified by sequencing the entire stTA2-cassette plus adjoining DNA sequences. Confirmation of the integrity of pPAC4-Cam-L065-stTA2.pA after recombination was done with XhoI digestion and Pulse-field gel electrophoresis (PFGE) analyses. The tTA-modified genomic *Tph2* sequence was separated from the PAC backbone by NotI digestion and subsequent preparative PFGE. The purified, linearized DNA fragment was microinjected into the male pronucleus of BDF1 mouse oocytes.

For functional analysis of Ptet-controlled gene expression, TPH2-tTA mice were mated to homozygous Ptet-nLacZ (NZL2) mice [15] to generate double transgenic TPH2-tTA/Ptet-nLacZ mice. For analysis of Ptet-controlled, Cre-mediated recombination, TPH2-tTA mice were mated to homozygous Ptet-Cre/R26R mice [12], [17] to generate triple-transgenic TPH2-tTA/Ptet-Cre/R26R mice.

All experimental procedures were approved by the local Animal Welfare Committee (Regierungspräsidium Karlsruhe 35-9185.81/G-107/09) and carried out in accordance with the local Animal Welfare Act and the European Communities Council Directive of 24 November 1986 (86/609/EEC).

### Doxycycline Treatment

Doxycycline (Dox) (Sigma) was dissolved in tab water supplemented with 5% Sucrose. For Ptet-controlled suppression of gene expression or Ptet-controlled, Cre-mediated gene deletion during embryonic development, 5 µg/ml Dox was administered via drinking water from the beginning of matings until birth. Mice with continuous Dox suppression until adulthood were given 5 µg/ml Dox in the drinking water from the beginning of matings until birth and 50 µg/ml Dox from P0-P60. Dox treatment in adult mice was always carried out with 50 µg/ml Dox. Activation of Ptet-controlled gene expression or Ptet-controlled, Cre-mediated gene deletion without Dox routinely lasted for 30 days.

### Non-radioactive *in situ* Hybridization

To generate a 700 bp cDNA tTA-template for antisense riboprobes we incorporated a T7 promoter into the antisense primer (tTA-Start: 5′-ATG TCT AGA CTG GAC AAG AGC A-3′, T7-tTA Stop: 5′-*GTA ATA CGA CTC* ATA GGG CAG CAG GCA GCA-3′). Plasmid pUHT 61-1 (Urlinger *et al.*, 2000) containing the full-length stTA2 cDNA was used for PCR. The PCR-product was extracted with phenol/chloroform. RNA in vitro transcription was performed using digoxigenin-labelled (DIG) UTP (Roche). After 2 h incubation, DNase was added to digest the DNA template. DIG-labelled UTP which was not incorporated into the RNA transcript was eliminated with NucAway Spin columns (Ambion). TPH2-tTA mice from each founder line were perfused with 4% paraformaldehyde (PFA) and postfixed for 8 h in 4% PFA. Prior to *in situ* hybridization (ISH), 50 µm coronal brain sections were cut on a vibratome and washed with 1×PBS and 1×PBS/0.1%Triton-X. Prehybridization of brain sections was carried out at 65°C for 1 h in hybridization buffer (25 ml Formamide, 0.5 ml Yeast tRNA [10 µg/µl], 5 ml DEPC-PBS, 10 ml 20×SSC, 5 ml Dextran 50%, 1 ml 50×Denhard, 3.5 ml DEPC-H2O) without the tTA-riboprobe. Afterwards the tTA-riboprobe was denatured at 95°C for 2 min and 300 ng/ml were used for hybridization in hybridization buffer for 18 h at 65°C. The next day, brain sections were washed with descending concentrations of SSC followed by two wash-steps with 1×Dig-solution (24.2 g Tris, 17.52 g NaCl, 148 ml 1 M HCL, 1260 ml DEPC-H2O) plus 4% BSA/0.1% Triton-X and one wash-step with 1×Dig-solution plus 2% BSA/0.1% Triton-X. Thereafter, the brain sections were incubated in 3×Dig-solution (72.6 g Tris, 52.56 g NaCl, 444 ml 1 M HCl, 1260 ml DEPC-H2O) with 1/750 anti-DIG-AP antibody (Dako) for 12 h at 4°C. The next day, brain sections were washed repeatedly with 1×Dig and 1×TBS. Finally, BCIP was added to a 1×TBS/MgCl solution for 10–30 min and the staining process was terminated with several 1×PBS washes. Brain sections were mounted on slides with Eukitt (Kindler GmbH, Freiburg, Germany).

### Histology

For X-Gal staining, adult mice were perfused with 4% PFA and postfixed with 0.2% Glutaraldehyde in lacZ fix solution (2 mM MgCl, 5 mM EGTA in 100 mM sodium phosphate, pH 7.3) at 4°C for 8 hours. Brains from embryos were fixed with 4% PFA at 4°C for one hour. 50 µm coronal brain sections were cut on a vibratome (Leica), washed with 1×PBS and stained with X-Gal staining solution (2 mM MgCl2, 5 mM K3[Fe(CN)6], 5 mM K4[Fe(CN)6] and 1 mg/ml X-Gal in 1×PBS) at 37°C for 1–4 hours and counterstained with Eosin (0.01 mg/ml 1×PBS).

### Immunohistochemistry

Adult mice were perfused with 4% PFA at 4°C. Brains were postfixed with 4% PFA at 4°C for 24 hours. 50 µm coronal sections were cut on a vibratome (Leica). Double-fluorescence IHC was performed on floating sections.

Brains from TPH2-tTA/Ptet-Cre/R26R embryos were fixed with 4% PFA at 4°C for 24 hours, dehydrated with sucrose (10%) for another 24 hours and frozen at −80°C. Coronal sections (20 µm) were cut on a kryostat (Leica CM 1950) and sections were mounted on tissue slides (Super Frost Ultra Plus, Menzel Gläser, Germany). Double-fluorescence IHC was performed in a humid chamber.

For IHC of the gut, TPH2-TA/Ptet-Cre/R26R mice (P21) were perfused with 4% PFA at 4°C. The entire body was postfixed with 4% PFA at 4°C for 24 hours and dehydrated with sucrose (10%) for another 24 hours and frozen at −80°C. 50 µm coronal section of the entire gastrointestinal (GI) tract were cut on a kryostat (Leica CM 1950) and sections were mounted on tissue slides (Super Frost Ultra Plus, Menzel Gläser, Germany). Double-fluorescence IHC was performed in a humid chamber. For tissue slide IHC, sections were first rehydrated with 1×PBS and blocked with 10% donkey serum in 1×PBS for 1 hour. For IHC with adult floating sections, coronal sections were first permeabilized with Triton X-100 (0.1%) for 30 min in 1×PBS at 4°C. Sections were then washed with 1×PBS (3×) and blocked with 10% donkey serum in 1×PBS for 1 hour. For both tissue slide and floating section IHC, primary antibodies were added to the blocking solution and incubated at 4°C overnight. After washing with 1×PBS the sections were incubated in blocking solution containing the secondary antibodies for 1 hour at room temperature. After final washes with 1×PBS, sections were mounted in Dako fluorescence mounting medium. The following primary antibodies were used: rabbit α-TPH2 (Dianova, 1∶5.000 in postnatal mice and 1∶2000 in embryos) and chicken α-β-galactosidase (Abcam, 1∶10.000). Secondary antibodies were AF488 donkey α-rabbit (Invitrogen, 1∶500) and Cy3 donkey α-chicken (Jackson Immuno, 1∶1000). Coronal vibratome sections were examined using a Nikon C1Si-CLSM confocal laser-scanning microscope (Nikon Imaging Center, BioQuant, Heidelberg, Germany). Embryonal and GI-tract sections were examined on a Leica TCS SP5 confocal laser-scanning microscope (ZI, Mannheim, Germany).

### Statistical Methods

Coronal slices of adult TPH2-tTA mice of each founder line were processed with dual-label fluorescent IHC detecting βgal and TPH2. Image stacks of all slices that showed TPH2 staining were acquired using a confocal laser-scanning microscope. The ratio of βgal +/TPH2+ neurons to all TPH2+ neurons was calculated separately for caudal, median and dorsal raphe nuclei. Confidence-bounds (CI) for expression efficacy in adult mice were calculated using the Clopper-Pearson method based on significance level 95.0% [76].
